# Vegetation and Climate Change during the Last Deglaciation in the Great Khingan Mountain, Northeastern China

**DOI:** 10.1371/journal.pone.0146261

**Published:** 2016-01-05

**Authors:** Jing Wu, Qiang Liu, Luo Wang, Guo-qiang Chu, Jia-qi Liu

**Affiliations:** Key Laboratory of Cenozoic Geology and Environment, Institute of Geology and Geophysics, Chinese Academy of Sciences, Beijing, China; Institute of Tibetan Plateau Research, CHINA

## Abstract

The Great Khingan Mountain range, Northeast China, is located on the northern limit of modern East Asian Summer Monsoon (EASM) and thus highly sensitive to the extension of the EASM from glacial to interglacial modes. Here, we present a high-resolution pollen record covering the last glacial maximum and the early Holocene from a closed crater Lake Moon to reconstruct vegetation history during the glacial-interglacial transition and thus register the evolution of the EASM during the last deglaciation. The vegetation history has gone through distinct changes from subalpine meadow in the last glacial maximum to dry steppe dominated by *Artemisia* from 20.3 to 17.4 ka BP, subalpine meadow dominated by Cyperaceae and *Artemisia* between 17.4 and 14.4 ka BP, and forest steppe dominated by *Betula* and *Artemisia* after 14.4 ka BP. The pollen-based temperature index demonstrates a gradual warming trend started at around 20.3 ka BP with interruptions of several brief events. Two cold conditions occurred around at 17.2–16.6 ka BP and 12.8–11.8 ka BP, temporally correlating to the Henrich 1 and the Younger Dryas events respectively, 1and abrupt warming events occurred around at 14.4 ka BP and 11.8 ka BP, probably relevant to the beginning of the Bølling-Allerød stages and the Holocene. The pollen-based moisture proxy shows distinct drought condition during the last glacial maximum (20.3–18.0 ka BP) and the Younger Dryas. The climate history based on pollen record of Lake Moon suggests that the regional temperature variability was coherent with the classical climate in the North Atlantic, implying the dominance of the high latitude processes on the EASM evolution from the Last Glacial Maximum (LGM) to early Holocene. The local humidity variability was influenced by the EASM limitedly before the Bølling-Allerød warming, which is mainly controlled by the summer rainfall due to the EASM front covering the Northeast China after that.

## Introduction

The last deglaciation is of great interest, because the climate in the Northern Hemisphere has gone through several distinct changes such as the LGM, Heinrich stadial 1 (H 1), the Bølling-Allerød (B-A) warm phases and the Younger Dryas (YD) cold periods [1,2]. Greenland ice cores provide the best expression of the climatic change during the last deglaciation. The temperature increase from average glacial to Holocene conditions indicated by the ice and gas isotope signal is approximately 15°C presented as the warming at the end of the Younger Dryas [3–5]. The further understanding of Dansgaard–Oeschger events and Heinrich events makes that the link between ice sheet behavior from Greenland and ocean–atmosphere temperature changes from North Atlantic sediments is recognized [6–8]. In the Asian Monsoon region, the δ^18^O records from Chinese and Indian stalagmites imply a weakening of the summer Indo-Asian monsoon during the YD and H 1, and stronger summer monsoon during warm period such as the B-A and early Holocene [9–11]. They implied that topical/subtropical Asian monsoon system is responsive to Northern Hemisphere summer insolation over the last two interglacial-glacial cycles [12]. In addition, some sedimentary records suggested different impact factors of climate change during the last deglaciation. For example, multi-proxy records from the Okinawa trough on the East Asian margin sea revealed that terrestrial warming controlled by high-latitude process lagged behind oceanic changes by 3–4 ka during the last deglaciation, being interpreted as low-latitude ocean dominance [13]. Magnetic properties and the titanium content from Huguangyan maar lake in sourthen China suggested an increase in winter Asian monsoon strength during the YD influenced by the intertropical convergence zone (ITCZ) [14]. The influences of impact factors from high-latitude process like the ice volume and low-latitude process like ITCZ and sea surface temperature (SST) to the climate change in the Asian Monsoon region are still controversial. In the northern limit of the East Asian Summer Monsoon (EASM), however, a few of records can extend to the LGM because most lakes formed after the last deglaciation [15–19].

The Great Khingan Mountain range is located in the northern boundary of the EASM. In biogeography, this area is a boreal-temperate forest transition zone in north-south direction as a reaction of temperature gradient, and a woodland-to-grassland transition zone in east-west direction as a response to monsoonal rainfall extension. The vegetation evolution in the region might be sensitive to the rapid climate changes. It is expected that the vegetation history recorded in this region could capture the regional monsoon variations in response to large climatic changes during the last deglaciation. Here, we report a high-resolution pollen record from Lake Moon with aims to understand how the regional monsoonal climate responded to the shift in glacial/interglacial modes.

## Materials and Methods

### Study site

The Great Khingan Mountain range forms a natural border between Inner Mongolia Plateau and Songliao Plain, which is constituted by a series of shallow hills arranged from northeast to southwest in the northeast China. The modern vegetation in the Great Khingan Mountain range varies from cold temperate conifer forest (Euro-Siberian taiga forest) in the north, to the temperate deciduous broadleaved forest in the southeastern slope. According to the precipitation or humidity gradient along east-west transect, the Great Khingan Mountain is a transitional zone from temperate deciduous broadleaved forest to forest-steppe. The dominant species is *Larix gmelinii* with other important species include *Picea koraiensis*, *Betula platyphylla*, *Quercus mongolica*, *Betula davurica*, *Populus*. The temperate deciduous broadleaved forest in the southeastern slope is characterized by *Quercus*, *Acer*, *Betula* and *Tilia*. The forest-steppe is distributed in the western slope of the Great Khingan Mountain. The steppe communities are dominated by *Stipa baicalensi*, *Aneurolepidium chinense*, *Filifolium sibiricum*, *Carex* spp., while the isolated forest patches are formed by the major species of *B*. *platyphylla*, *B*. *dahurica*, *Q*. *mongolicus* and *L*. *gmelinii*. The steppe vegetation increased in the west and composed mainly of *Stipa grandis*, *Stipa krylovii*, *Cleistogenes squarrosa*, and *Artemisia frigid* with minor components of *Agropyrum cristatum*, *Aneurolepidium chinense*, *Carex duriuscula*, *Artemisia* spp. and *Achnatherum splendens* [20].

**Fig 1:**
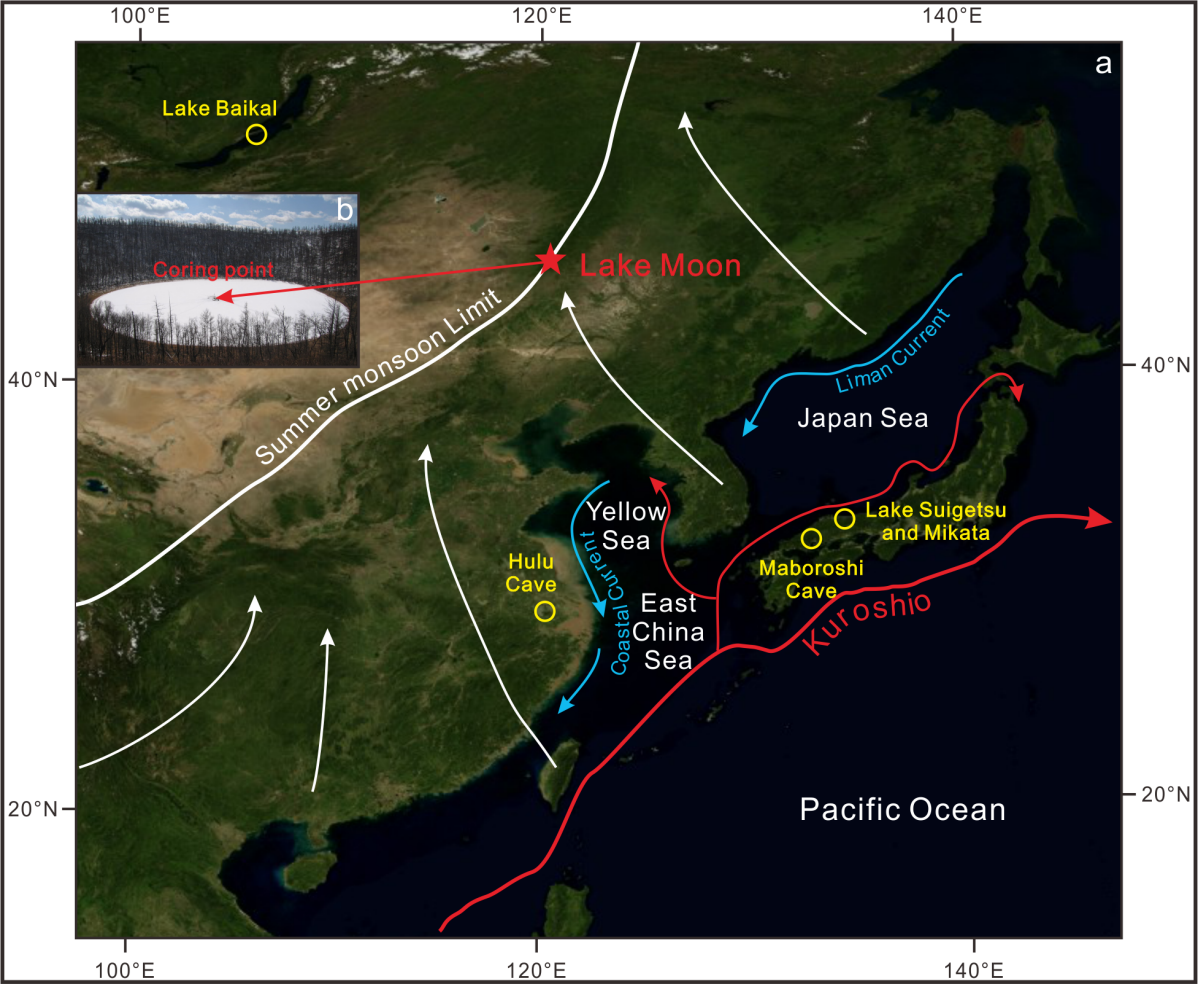
Locations of study site and other paleoclimate records in the East Asia. (map modified from NASA; http://earthobservatory.nasa.gov/) Locations of Lake Moon, Baikal (52°05′N, 105°52′E), Suigetsu (35°35′N, 135°53′E) and Mikata (35°33′N, 135°54′E), and Hulu (32°30′N, 119°10′E) and Maboroshi (34°39′N, 133°13′E) caves with atmospheric circulation in summer. Black arrows show seasonal dominant wind vectors and the red arrow points to the location of coring point of Lake Moon.

We selected a small closed crater lake, Lake Moon (47°30.36′N, 120°51.99′E, 1190m a.s.l), as study site. This lake is located in the middle of the Great Khingan Mountain range (Fig 1), in Chaihe Town, Zhalantun City, Inner Mongolia. The diameter of the lake is 220m with the maximum water depth of 6.5m and the volcanic cone formatted in Middle Quaternary [21].

**Fig 2:**
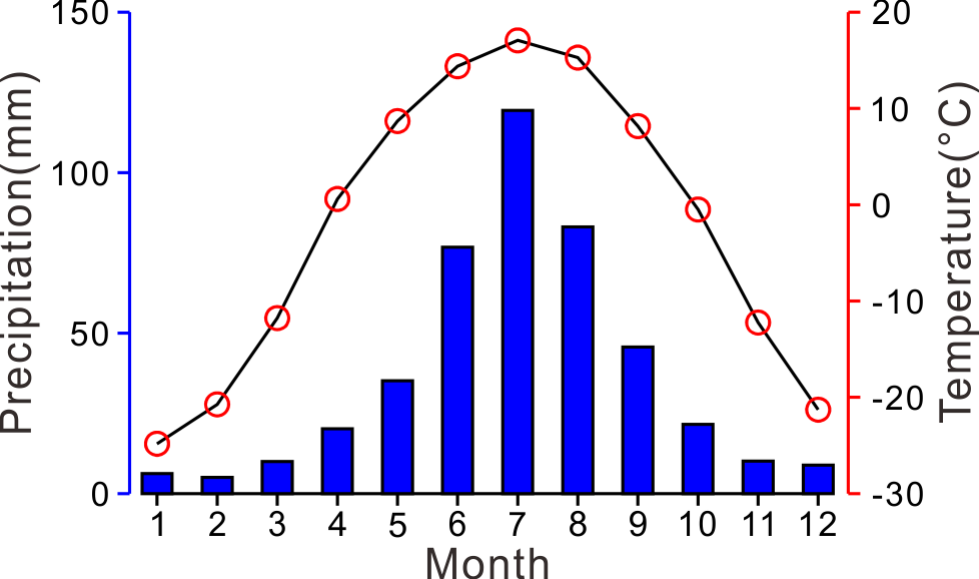
Averaged monthly precipitation (bars) and air temperatures (circles) in Aershan during AD 1982–2011.

Present climatic conditions show a pronounced seasonality with a warm and humid summer and a cold and dry winter. The mean annual temperature during AD 1982–2011 is -2.3°C and the mean annual precipitation is 442 mm, in which 63% precipitation occur in summer season (June to August) (Fig 2). Lake Moon is ice-covered from the end of October to the start of May next year.

The modern vegetation in the catchment of Lake Moon is composed of a deciduous broadleaf-conifer mixed forest. The forest is dominated by *L*. *gmelinii*, *B*. *platyphyll* and *B*. *dahurica*, with a shrub layer of *Corylus heterophylla*, *Rhododendron dauricumand*, *Lespedeza bicolour* and *Pyrola calliantha*, and the herbaceous species including *Iris uniflora*, *Deyeuxia langsdorffii*, *Artemisia integrifolia*, *Artemisia gmelinii*, *Artemisia tanacetifolia*, *Fragaria orientalis*, *Sanguisorba officinalis*, *Vicia unijuga* and *Saussurea ciliaris*. *Salix* spp. and *Spiraea salicifolia* shrubs are distributed sporadically about 25m away from the lakeshore. The hygrocolous communities around the lake are dominated by *Deyeuxia angustifolia*, *Deyeuxia langsdorffii*, *Carex appendiculata* and *Carex rhynchophysa*.

### Ethics Statement

All necessary permits were obtained for the described field work and were granted by the local government of Inner Mongolia. The field work did not involve endangered or protected species.

### Sediment and chronology

Three over-lapping piston cores were recovered from the center of Lake Moon in the early spring of 2007. The sediment is presented as dark brown finely laminated gyttja from 0 to 590 cm; brown organic-rich laminated clay from 590 to 732 cm; and grey clay from 732 to 886 cm. Twenty-one AMS ^14^C dates were obtained from Poznan radiocarbon Laboratory, of which 15 samples above 610 cm are plant macro remains (leaves, fructifications and seeds); and 6 samples below 610 cm are bulk sediment, all age data have been published before [22].

### Pollen analysis

The cores were split in half longitudinally and subsamples for pollen analysis were taken at intervals of 2 cm from 546 cm to the bottom of the core. *Lycopodium* spores were added in order to calculate the pollen concentration (grains g^-1^). One hundred and seventy-one subsamples were prepared with alkaline and acidic treatments [23], followed by ultrasonic sieving through 7 mm mesh. Pollen identification was under optical microscope at ×400 magnification with the aid of publications on pollen atlases and keys [24,25]. More than 400 pollen grains were counted for most samples, varying from 357 to 890. Pollen percentages were calculated for each taxa by using the sum of terrestrial pollen. To detect the influence of environmental impact factors on pollen-based vegetation evolution from the sediment sequence of Lake Moon, the Principal Componenta Analysis (PCA) was used as ordination techniques performed by SPSS version 19.0.0 software. Nine pollen taxa whose percentages are higher than 5% in any sample were used for PCA.

## Results

**Fig 3:**
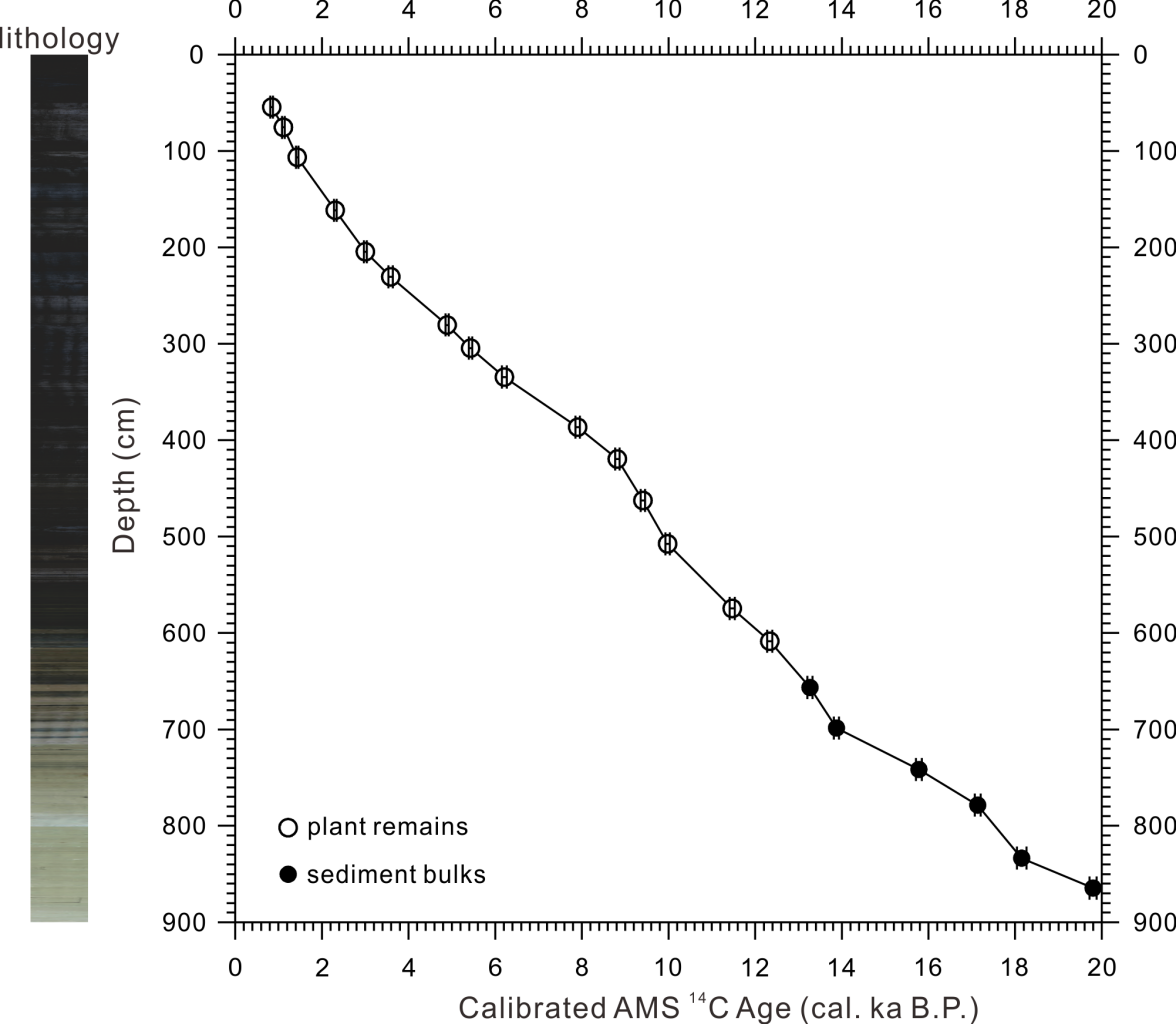
Age-depth plot of the Moon Lake sequence. The calibrated AMS ^14^C dates using CALIB 6.01 [26,27] are shown with 2 sigma error bar (adapted from Fig 2 of [22]).

All the ^14^C ages have been calibrated to calendar age by CALIB6.01 (http://calib.qub.ac.uk/calib/) program using IntCal09 database [26,27] (Table 1). A depth-age plot of the Lake Moon is shown in Fig 3. The ages increase monotonically with depth and suggest no significant sedimentary disturbance and a rather stable sedimentation rate. The smooth secession of fossil plant and bulk organic ^14^C ages through depth suggests that the old carbon effect in this lake could be neglectable.

**Table 1:** List of radiocarbon and calibrated ages of sediment core from Moon Lake (all age dates listed in this table are from previous publication [22]).

Samples			Radiocarbon age	Calibrated age	
Depth(cm)	Material	Lab. code	AMS ^14^C (yr BP)	cal a B.P.(2σ-range)	cal a B.P.(median)
507–508	leaves	Poz-27133	8,880±50	9,777–10,182	9,980
574–575	aquatic weed	Poz-27134	9,960±60	11,239–11,701	11,470
608–609	seeds	Poz-27135	10,450±60	12,116–12,560	12,338
656–657	mud	Poz-27220	11,400±60	13,134–13,396	13,265
698–699	mud	Poz-27138	12,010±60	13,729–14,023	13,876
741–742	mud	Poz-27139	13,040±70	15,171–16,387	15,779
778–779	mud	Poz-27140	14,030±70	16,841–17,432	17,136
833–834	mud	Poz-27221	14,870±110	17,770–18,531	18,150
864–865	mud	Poz-27141	16,640±80	19,480–20,114	19,797

**Fig 4:**
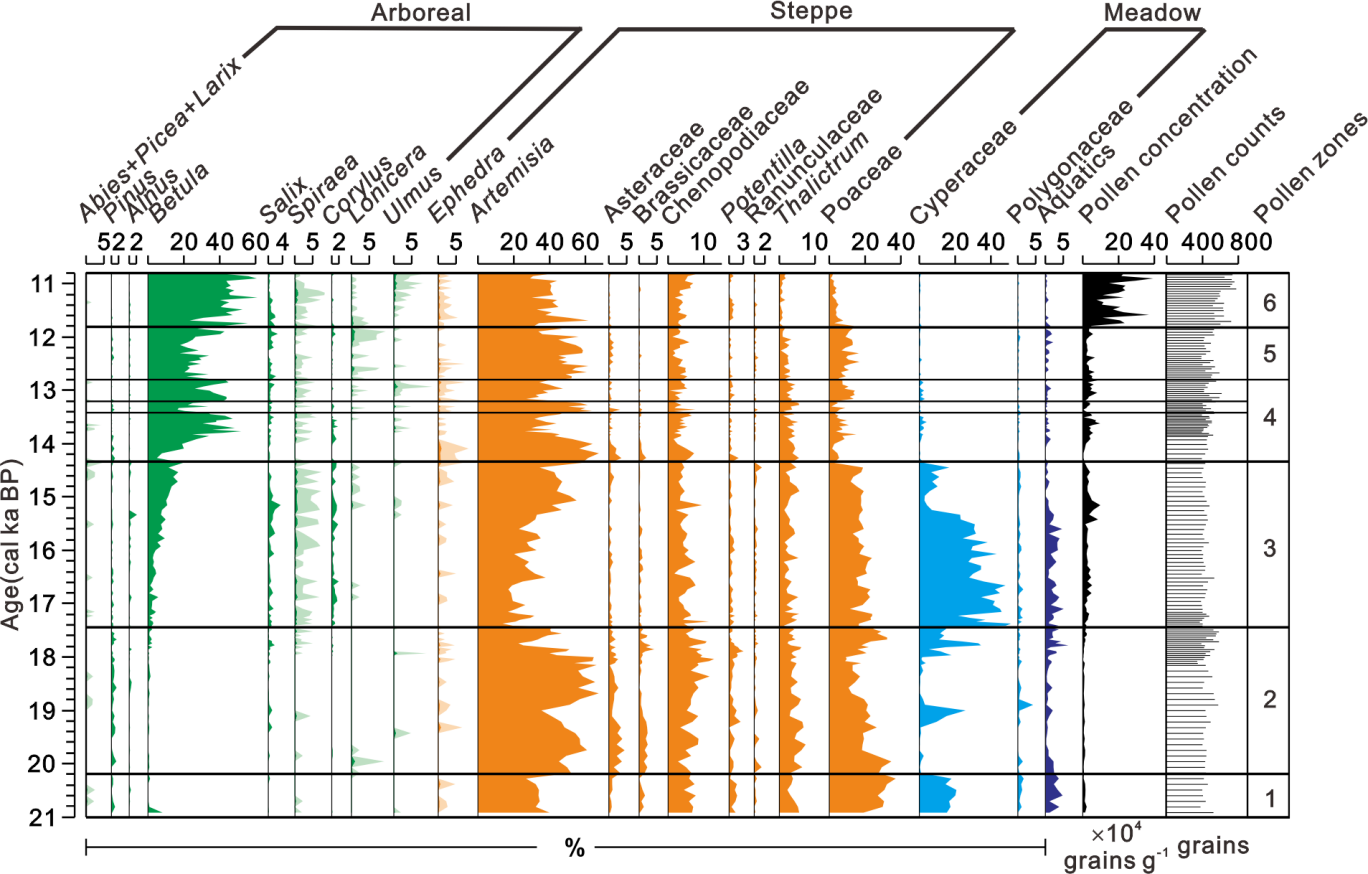
Simplified pollen percentage and concentration diagram of Lake Moon. Exaggeration (×10) is indicated by light-colored shading (adapted from Fig 2 of [29]).

A total of 35 arboreal taxa, 48 herbaceous and fern taxa were identified from the sediment sequence of Moon Lake. Six pollen assemblage zones were recognized based on the characteristic of arboreal and herbaceous pollen percentage and pollen concentration referred to the CONISS statistical method [28] against the chronology presented above (Fig 4).

Zone 1 (886-874cm, ~ca. 20.9-~20.3 ka BP): This zone is characterized by the dominant herbs (86.8–99.5%), *Artemisia* (26.7–40.0%), Poaceae (19.1–37.1%), Cyperaceae (14.6–20.7%), and abundant Chenopodiaceae and *Thalictrum* with low pollen concentration (5.2×10^3^−21.7×10^3^ grains g^-1^). The coniferous (0.2–1.0%) and deciduous broad-leaved (0–8.5%) taxa are sparse. In contrast, the percentage of aquatic taxa is relatively high contributed from *Myriophyllum* (0.8–4.8%).

Zone 2 (874-796cm, ~ca. 20.3-~17.4 ka BP): This zone is also dominated by herb components (94.4–99.2%), mainly of dry steppe taxa, *Artemisia* (22.5–67.4%), Poaceae (8.9–34.7%), Chenopodiaceae (2.7–12.9%), and also some meadow taxa like Cyperaceae (0–34.5%) and *Thalictrum* (2.3–7.4%). There is a peak of Cyperaceae with a sharp decrease of *Artemisia* between 19.0–18.8 cal. ka BP It still has low percentage of coniferous (0–2.3%) and deciduous broad-leaved (0–3.9%) pollen and low pollen concentration (2.4×10^3^−22.7×10^3^ grains g^-1^).

Zone 3 (796-710cm, ~ca. 17.4-~14.4 ka BP): This zone is marked by high alpine meadow taxa mainly of Cyperaceae (2.9–51.2%) and increasing of *Betula* (0–16.8%), at the expense of steppe taxa like *Artemisia* (13.7–55.1%). Pollen component still dominated by a variety of herbs including Poaceae (11.5–24.0%), Chenopodiaceae (1.8–11.4%), and *Thalictrum* (1.2–5.6%) besides the two taxa mentioned above. The pollen concentration (10.9×10^3^−98.8×10^3^ grains g^-1^) increases slightly with the first sign of the increasing percentage of trees.

Zone 4 (710-634cm, ~ca. 14.4-~12.8 ka BP): It features relative high and various deciduous broad-leaved components as *Betula* (5.0–52.0%). Reduced herbs components include *Artemisia* (26.1–67.6%), Poaceae (1.2–15.2%) and Chenopodiaceae (0.5–7.6%). Pollen concentration is 3.9×10^3^−97.0×10^3^ grains g^-1^ in this zone.

Zone 4 can be divided into 3 subzone based on the percentage of trees and herbs pollen, mainly of *Betula* and *Artemisia*. 4a is characterized by the increase of *Betula* (5.0–52.0%); 4b is marked by decrease of *Betula* (16.4–33.7%) and increase of *Artemisia* (50.4–63.7%); 4c has relative high percentage of *Betula* (26.1–44.9%) and low percentage of *Artemisia* (26.9–47.4%).

Zone 5 (634-590cm, ~ca. 12.8-~11.8 ka BP): This zone features relative low deciduous broad-leaved component (17.7–42.7%) mainly *Betula* (17.7–42.7%) accompanied with relative high herbs component includes *Artemisia* (31.4–61.7%), Poaceae (5.2–17.0%) and Chenopodiaceae (1.2–6.0%) compared to last zone. The low pollen concentration (4.3×10^3^−97.0×10^3^ grains g^-1^) associated with rise of herbs indicates the steppe component expands.

Zone 6 (590-544cm, ~ca. 11.8-~10.8 ka BP): It is characterized by the high pollen concentration (77.1×10^3^−0.4×10^6^ grains g^-1^). This zone was dominated by herbs (37.3–75.6%) and deciduous broad-leaved (23.4–61.4%) component including steppe taxa like *Artemisia* (29.1–61.8%), Chenopodiaceae (1.9–8.1%) and broad-leaved forest taxa as *Betula* (23.2–61.2%).

## Discussions

### Vegetation and climate changes from the LGM to early Holocene

Prior to 20.3 ka BP, pollen percentage of herbs are dominant including highest pollen percentage of Poaceae for the entire core and high percentages of Cyperaceae, Chenopodiaceae, *Artemisia* and *Thalictrum*. Poaceae and Cyperaceae are common compositions of modern pollen records from steppe, forest steppe, meadow and wetlands communities surrounding study area, whose pollen are under-represented [30,31]. Generally the percentage of them will not exceed 10% from steppe and forest steppe [32], which probably implies that the pollen assemblage before 20.3 ka BP varies from that of the modern steppe and forest steppe nearby [32]. According to the previous modern pollen studies on the Tibetan plateau [33–36], the pollen assemblage before 20.3 ka BP may be similar to the Tibetan alpine steppe with lower percentage of Cyperaceae than alpine meadow [33,34,36]. The Tibetan alpine steppe grows in the region where the mean annual temperature varies from -5.2°C to 0.1°C with -3.4°C as mean value, and the mean annual precipitation ranges from 161mm to 498mm with 328mm as mean value [34]. It suggests a colder and drier climate during this period than present.

During the period 20.3–17.4 ka BP, pollen percentage of herbs are still very high, and dominated by highest pollen percentage of *Artemisia*, Chenopodiaceae, and *Thalictrum* throughout the whole sediment sequences and the moderate decrease of Poaceae. The assemblage indicates that the subalpine meadow or steppe retreated and the dry steppe expanded, implying that the climate became slightly warming and drying.

During the interval from 17.4 to 14.4 ka BP, subalpine meadow recovered and the dry steppe shrank around the study site, as indicated by high Cyperaceae and low *Artemisia* percentage. Different from the period before 20.3 ka BP, arboreal pollen percentage shows a visibly increase during this interval accompanied by expansions of pioneer forest components *Salix* and *Corylus* [37] and noticeable increase in pollen concentrations. It suggests a milder and slightly humid condition than those of previous stages.

A substantial change of the vegetation took place between ~ca. 14.4 and 13.4 ka BP (Subzone 4a). Arboreal elements became prominent since this interval, as the percentage of *Betula* increased at the expense of *Artemisia* which suggests a temperate broad-leaved forest expanded during this period and the vegetation was transiting from subalpine meadow to forest steppe. Otherwise, the lithology changes from white-gray clay (LG) to brown clay and black organic-rich clay with discontinued lamina, indicates that the lake water level increased probably due to increasing moisture or precipitation and suggests that the EASM became stronger. However, some cold climate events have possibly occurred, such as 13.6, 13.4–13.2 (Subzone 4b), 13.0 and 12.8–11.8 ka BP (Zone 5), implied by high proportion of herbs and lower proportion of trees suggesting the weakening of the EASM.

In the early Holocene from 11.8 to 10.8 ka BP, the vegetation type was still forest steppe while the extremely high pollen concentration throughout this interval suggests that the climate became significantly warmer and moister. The warmer and moister climate can be also inferred from the lake environment; the continued black-white lamina indicates a deep lake status. Moreover, the temperate deciduous broadleaved trees such as *Betula* and *Ulmus* increased at the expense of herbs like *Artemisia*, Poaceae and *Thalictrum*, which is the result of the intensification of the EASM.

### Temperature and moisture index estimated from pollen taxa groups

**Fig 5:**
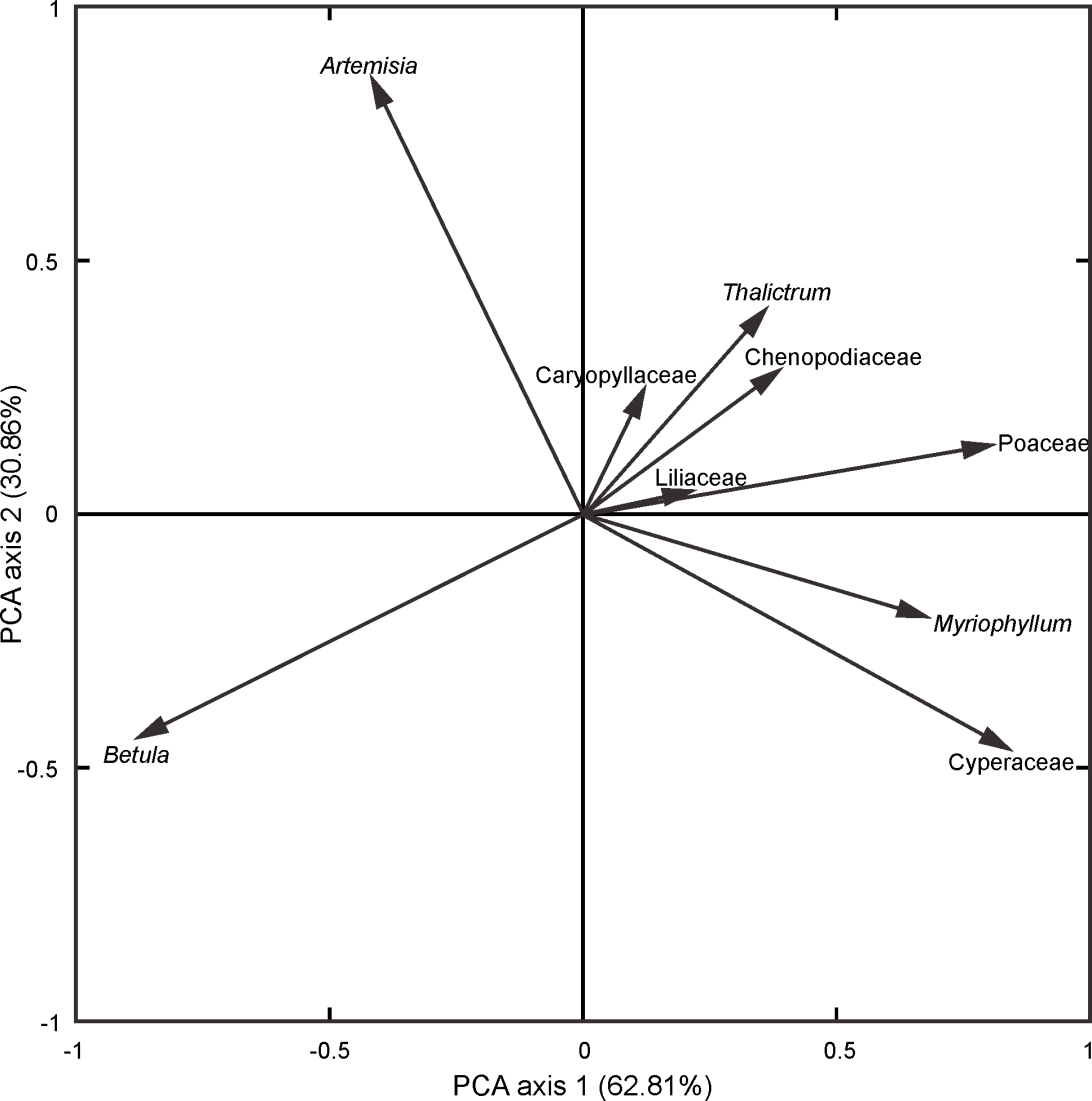
PCA ordination of nine pollen taxa with percentages >5% in any sample of pollen assemblages from Lake Moon.

The distribution of vegetation types is primarily determined by both temperature and moisture, therefore temperature and moisture could be semi-quantitatively estimated based on the ratios between groups of pollen taxa represented different temperature and moisture conditions [38–40]. As mentioned above, the geographic distribution of modern vegetation types in the study area shows obvious gradients of temperature and moisture. The decline of temperate deciduous broad-leaved species pollen content and the increase of cold conifer species reflect a decreasing temperature. Moreover, the even colder climate is implied by the existence of alpine meadow, which only distribute in high mountains in China under cold condition today. The previous studies showed that during the LGM, meadow dominated by Cyperaceae occupied the areas covered by cold conifer forests or even temperate forests today [41,42]. Thus, the increasing of alpine meadow pollen content could imply the dramatic decrease of temperature. Otherwise, the increasing ratio of steppe pollen content represents the steppe expanding responded to the reduction of humidity. This hypothesis also has some sympathy with the results of PCA of the pollen taxa, whose percentages are higher than 5%. As shown in Fig 5, PCA axis 1 separates broadleaved arboreal *Betula* and steppe *Artemisia* from meadow and other herbaceous taxa, whereas PCA axis 2 distinguishes steppe taxa from arborous and meadow taxa. This implies that the first principle component might mainly represent temperature with the taxa scores on the axis 1 positively related to cold conditions, and the second principle component might mainly represent humidity with the taxa scores on the axis 2 positively related to dry conditions. The first two axes capture most variance within the data set, which implies that the increasing of temperature is considered as the main factor of vegetation change from alpine meadow to forest steppe and the decreasing of humidity cause the vegetation change into steppe.

Based on above-mentioned, we tentatively define the ratio of temperature broadleaved and shrubs taxa to alpine meadow taxa as temperature index (T) and the ratio of arboreal pollen percentage to steppe taxa percentage as a moisture index (M). The detailed taxa for calculations of indices are listed in Table 2. The similar methods have been used to reconstruct temperature and moisture variations in the northern Mongolian Plateau and Baikal region where the vegetation types distribute along the similar gradients as the study area [38–40].

**Table 2:** Five pollen taxa groups (S1-S5) used for calculation of temperature index (T) and moisture index (M).

Group	Vegetation types	Pollen taxa included
S1	Steppe taxa	*Artemisia*, Poaceae, Chenopodiaceae, *Thalictrum*, *Sanguisorba officinalis*, Asteraceae, Brassicaceae, Caryopyllaceae, Lamiaceae, *Plantago*, Ranunculaceae undiff., *Ephedra*, Apiaceae, Scrophulariaceae, Convolulaceae, Leguminosae, Sedum-type, *Tamarix*, *Nitraria*, Liliaceae, *Humulus* and selected further NAP
S2	Temperate broadleave taxa	*Betula*, *Alnus*, *Ulmus*, *Juglans*, *Carpinus*, *Pterocarya*, *Quercus*, *Acer*, *Tilia* (AP)
S3	Shrubs taxa	*Salix*, *Corylus*, *Spiraea*, *Hippophae* and selected further AP
S4	Taiga taxa	*Pinus*, *Juniperus*, *Larix*, *Picea*, *Abies*, *Sambucus*, *Lonicera* (AP)
S5	Alpine meadow taxa	Ericaceae, Cyperaceae, *Saxifraga*, *Rumex*, *Potentilla*-type, *Oxyria*, Polygonum undiff., Gentianaceae, *Pedicularis* (NAP)
Index	Vegetational indices	Calculation
T	Temperature index	T = (S2+S3)/S5
M	Moisture index	M = (S2+S3+S4)/S1

### Comparison of the pollen-based temperature index and δ^18^O from Greenland ice core

**Fig 6:**
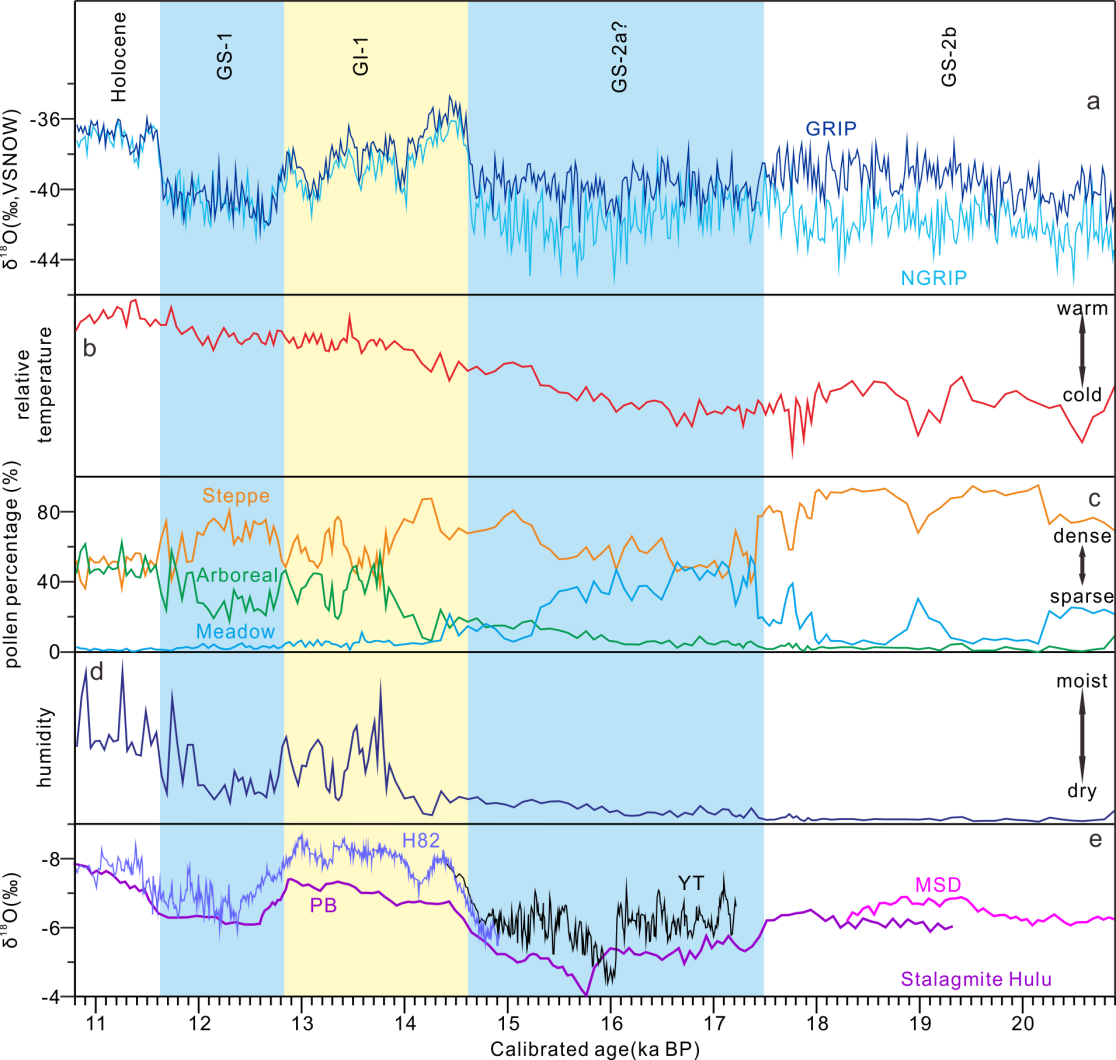
Comparison of pollen records with the δ^18^O records from Greenland ice core and Chinese stalagmite. (a) The δ^18^O records of the Greenland ice cores [47,48] (the light blue line is NGRIP δ^18^O profile, the dark blue line is GRIP δ^18^O profile), (b) the common logarithm transformation of pollen-based Temperature index (to outweigh strong variations caused by percentage maxima of *Betula* and Cyperaceae), (c) pollen percentages of arboreal (green line), steppe (orange line) and meadow (blue line) taxa, (d) pollen-based Moisture index, (e) the stalagmite δ^18^O records from Hulu cave [10].

For better understand the vegetation history presented here we correlated the pollen-based temperature series to the NGRIP δ^18^O record (Fig 6). The coldest temperature appeared at ca. 20.6 ka BP (Zone 1), subsequently with a temperature increase. The Zone 1 and 2 stage might correspond to Greenland Stadial GS-2b, characterized as a relative mild period in the last glaciation. The first notable warming since ca. 21 ka BP indicated by the vegetation change around the Lake Moon occurred in the beginning of the Zone 2 stage at ~20 ka BP. It provided vigorous evidence for the terrestrial signals of the warming in East Asia. It used to be considered that the terrestrial signals of the last deglacial warming evidenced by the paleovegetation changes began ca. 15 ka BP in East Asia as the onset of the Bølling warm phase, which is ca. 3–4 ka behind the warming of SST in the East China Sea and tropical western Pacific Ocean [13]. However, the increasing trends of the temperature implied by vegetation change surrounding Lake Moon since ca. 20 ka BP seems to be a near-synchronous response to the variations of the δ^18^O curve suggested temperature increasing occurred ca. 20–19 ka BP in the North Atlantic region [43–45]. This warming is also implied by the decreasing percentage of cryophilous pollen taxa like Ericaceae and climbing trend of the δ^18^O curve in other paleoclimate records of the East Asia [10,46] (Fig 6), when the summer insolation is increasing. This increasing of temperature in the East Asia at ca. 20 ka might be the response to the worldwide insolation increasing in summer.

The Zone 3 stage is related to GS-2a, which is not encouraged to use, as the feature of GS-2a is not consistent between ice records. However, the punctuated decreasing of temperature during the Zone 3 stage documents this cold event from ca. 17.4 to 14.4 ka BP. The coldest period from 17.2 to 16.6 ka BP is nearly synchronous with the record of ice-rafted detritus in North Atlantic region as H1 [43].

The obvious increasing of temperature marked the onset of the warm event (Zone 4) at 14.4 ka BP, which corresponds to the start of Greenland Interstadial GI-1. Two temperate/warm phases, counterparts of Bølling (Zone 4a) and Allerød (Zone 4c) are characterized by higher or increasing proportions of temperate deciduous broadleaved trees to distinguish. They are separated by distinct and short cold phases with relative high herb contents, as Older Dryas (Zone 4b).

The decrease in relative temperature calculated based on the pollen data of Lake Moon suggests a cold and dry event begins at ~12.8 ka BP. This major climate deterioration represented by Zone 5 is comparable with the Younger Dryas/GS-1 in the North Atlantic region, whose amplitude in East Asia appears much smaller than that in the North Atlantic region [49], which implied by the ratio change between arboreal and steppe pollen taxa within the forest-steppe vegetation type. The temperature cooling of this cold reversal is obvious in east coastal area along the Pacific Ocean [49,50], but is even ambiguous in inland region like Lake Baikal region [38]. The amplitude of the YD in the North Atlantic region is almost as large as that of glacial–interglacial cycles while the amplitude of this pan-hemispheric Late Glacial cold event decreases in other regions, which implies that the meltwater pulse is the most likely cause of the YD event [51]. The Zone 6, following the YD event, reveals that the temperature raises again in the early Holocene near-synchronous with the increasing δ^18^O values from Greenland ice core.

The errors of age model based on ^14^C data and the gradualness of vegetation response to climate change make it impossible to demonstrate absolute synchronous between the pollen records from Lake Moon and δ^18^O values from Greenland ice core. However, it is clear that cold and warm events found in the Lake Moon sediments can be roughly matched with corresponding climate events in ice cores in North Atlantic regions and Greenland, which have been proven to be controlled by the insolation of Northern Hemisphere and the meltwater pulse [52,53]. If the deduction goes further, the signal of temperature change in East Asia controlled by EASM comes from high latitude region of the Northern Hemisphere.

### Comparing the pollen-based humidity index with other East Asia Monsoon records

Distinctly different trends are observed in relative temperature and humidity curves of Lake Moon (Fig 6). The driest climate lasted from 20.3 to 18.0 ka BP which is a relative mild period of temperature. After that, the humidity kept increasing until 12.8 ka without drought which is supposed to arise from H1-like event. The climate recovered from the drought occurred during YD-like event to moist in early Holocene. It demonstrates that there is inverse relationship between humidity and temperature in the study region during the LG, while after B-A the humidity and temperature show the same change pattern. The humidity curve of Lake Moon is also inconsistent with the other pollen-derived precipitation records of the lakes and δ^18^O records of caves from typical East Asian monsoon region with lower latitudes before B-A warming [54–56]. The inconsistency between the records before B-A warming could be explained in the following discussion on the evolution of EASM.

The previous studies show that the sea level rises 20-30m during the B-A warming [57–59]. The low sea level makes a large area of modern marginal sea exposed, which imply that the coastline is much far away from the study site before that time. The spatial scale of the EASM circulation system before B-A warming is smaller than modern monsoon, as the EASM could not move northward to study area before B-A warming. The summer rainfall is not linked to the intensity of EASM that time, and the regional humidity is mainly derived from snow melt in response to Northern Hemisphere summer insolation as concluded by previous studies from regions with higher latitudes [60,61]. The low evaporation capacity caused by low temperature makes the effective soil moisture relative high, which causes H1-like event as a cold and slightly wet episode. Therefore, it is possibly that the weak EASM has only a limited influence over the Lake Moon before B-A warming considering location of the lake.

Different from H1-like event, YD-like event is suggested as a cold and dry event from Lake Moon occurring at ca. 12.8 ka BP. The drought during this period is consistent from coast to inland [38,49,50], which implies that the impact of YD event is not only diffused by the thermohaline circulation of the ocean, but also by the continental-oceanic interaction like the EASM. The cooling SST caused a weakened strength of EASM, a shrink of the area affected by the water vapor transport originated from the Pacific Ocean and the drought appeared in the northwest margin of EASM. The difference presentation of humidity between the H1-like and YD-like cold events in East Asia state that the intensification of EASM occurring on B-A warming is one of the most important terminations of EAM system since LGM. The broad synchroneity of the late glacial and YD event, based on the comparison of the palynological evidences observed in this study and Sihailongwan records with the oxygen isotope signals from speleothems in Eastern China and also from the Greenland ice core suggests that the climate between the East Asia and the North Atlantic region is teleconnected since the B-A warming [16,18]. Changes in the precipitation of EASM on millennial to centennial scales in northeast China might be related to ocean–atmosphere interactions in the tropical Pacific linked to the North Atlantic region by the thermohaline circulation [62]. This implies that the climate change in the Great Khingan Mountain range has a close link to the North Atlantic through the thermohaline circulation since B-A warming.

## Conclusions

The pollen record from Lake Moon in the Great Khingan Mountain, Northeast China covers a time interval from 20.9 ka to 10.8 ka BP. It reveals six significant shifts in the vegetation: alpine meadow between 20.9 and 20.3 ka, dry steppe from 20.3 ka to 17.4 ka, alpine meadow again in the period of 17.4–14.4 ka and forest steppe since 14.4 ka within an expansion of steppe during 12.8–11.8 ka.

The vegetation changes and reconstructed temperature and humidity index from the Lake Moon show distinct changes such as H1, B-A warming and YD events recorded in the North Atlantic. The onset of first deglacial warming of the study area was at ~20.3 ka BP, which synchronous with the decreasing of the global ice volumes and rising of the sea level. This warming trend is interrupted by H1-like event, which suggests that the shutdown of thermohaline circulation has a significant effect on the EASM. The B-A warming after H1-like event implies a drastic warming and wetting of the deglacial climate under strong EASM influence. The following YD-like event suggests declines of temperature and humidity caused by the weakening of EASM, while the temperature and humidity rise further in early Holocene because of the intensification of EASM once again.

The pollen-based temperature reconstruction in the study area suggests that the change of ice volumes in high latitude region might play an important role, while the humidity could mainly be affected by the ocean conveyor belt circulation, teleconnected with the North Atlantic region after B-A warming.

## Supporting Information

S1 Table Data of pollen percaentage (%), concentration (grains g^-1^) and counts (grains) from Lake Moon. (XLS)
